# Modeling of the high-performance PLD-based sectioning method for classification of the shape of optical object images

**DOI:** 10.1186/2193-1801-2-692

**Published:** 2013-12-27

**Authors:** Leonid Tymchenko, Mycola Petrovskiy, Natalia Kokryatskaya, Volodymir Gubernatorov, Yuriy Kutaev

**Affiliations:** Department of Telecommunication Technologies and Automatics, State Economy and Technologies University of Transport, 19 Lukashevich Street, Kyiv, 03049 Ukraine; KIA Systems, Moscow, Russia

**Keywords:** Image processing, Laser systems, PLD(FPGA)

## Abstract

An analysis of the sectioning method to control a shape of the laser beam spot is conducted in this paper. A possibility is discussed to use a form factor for solving the problem raised, and the experimental study of the sectioning method and the form factor for control of the shape of the laser beam spot was conducted. A PLD-based technical implementation of the method was realized.

**Electronic supplementary material:**

The online version of this article (doi:10.1186/2193-1801-2-692) contains supplementary material, which is available to authorized users.

## Introduction

Rapidly growing requirements to modern computational media encourage development of new intelligent methods of information transfer and processing. Rigid requirements to real-time information processing systems force scientists to regularly create and upgrade data transfer systems. Today most internet channels cannot provide data exchange of the required quality between such systems, which, in turn, results in the congestion of those channels and formation of so-called digital bottlenecks. A possible solution of the problem of transfer of large volumes of information is to use a fiber-optic cable, but laying such cable is rather expensive, even on short distances. At the same time, this problem can be solved through application of the laser-based technologies (Khosrofian and Garetz, 1983; Kozhemyako et al., 2003; Basov et al., 1998), one of the most promising models of information transfer for the near future. In this case, for instance, tons of full-length films and virtual worlds could be transferred to any part of the globe in a blink of an eye.

Most satellites transmit information, such as TV programs, by means of microwave radiation, while laser-based information transmission could be hundreds of times faster, which, in turn, will considerably increase the carrying capacity of the channel.

The laser-based information transmission requires that both a satellite and a receiving unit (RU) were located in a certain position. A position of the RU lens, whose diameter is only several centimeters, must be adjusted to one thousandth degree, otherwise the information transmission will not happen.

During the process of tracing a satellite by a receiving unit, one of the main tasks is to classify a shape of the laser beam spot image, namely, its geometrical characteristics distorted by turbulence and air masses.

To solve a problem of classification and increase an accuracy of determination of the spot object center (Mana et al., 2001; Orlov and Neverova, 2003) by means of the maximal usage of its informational characteristics, frames of the series of laser beam spot images should be classified in order to filter the laser route from significantly noise-distorted images.

Known methods of analysis of the optical object shape, for instance described in (Magnes et al., 2010; Wright et al., 2011; Gannot et al., 2002), due to complexity of operations performed do not allow estimate their shape properly with simple computer aids. That’s why image processing using the PLD is particularly relevant in our time (Hartmann et al., 2008; Chien and Chen, 2008).

A goal of the work is to solve a scientific problem of developing a computationally simple and therefore high-performing classification method of surfaces of laser beam spot images with its further modeling using the PLD.

## The sectioning method for the real-time control of the shape of the beam spot surface

When realizing subsystems of image control and processing in such devises as laser locators or laser transfer system, a number of requirements arises that influence a choice of the method and processing instruments:

1. A unit should be low-weighted, small-sized, and have low energy consumption.
2. Processing should be conducted in the real time operation mode.

All that imposes certain restrictions on the choice of the algorithm and time of its execution. One of the methods allowing to conduct image processing and classification with acceptable parameters is a sectioning method for real-time control of the shape of the beam spot.

### The approximate classifying function

A traditional way of solving a problem of control of the shape of laser beam spots includes formation of the beam spot image *В*(*х, у*) on the photosensitive surface of the photo receiver and its further transformation into a signal *U*(*х, у*)*.* An amplitude of this signal in each point of the expansion with coordinates (х, у) corresponds to the intensity in *В*(*х, у*), i.e. *U*(*х, у*) ↔ *В*(*х, у*). Then the signal *U*(*х, у*) is being compared with a reference signal *W*(*х, у*) for all points of signal expansion. Signals *U*(*х, у*) constitute certain surfaces that may differ by type, relative scale coefficient, vector of the relative coordinate matching, and relative turning angles in the three-dimensional space. That is why comparison of those surfaces must be conducted taking into consideration all possible situations, which requires a huge amount of calculations and is difficult for the real-time realization.

**Figure 1:**
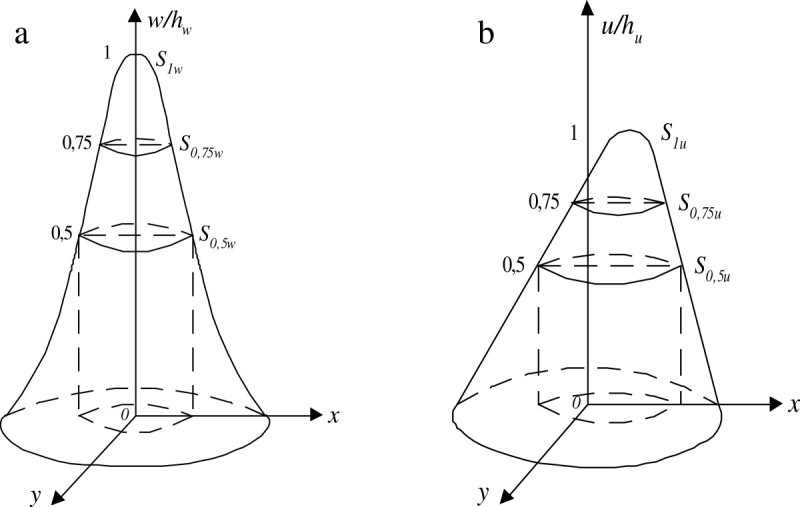
**Signals of the laser beam spot image. (a)** Reference image and **(b)** current image.

In practically important situations, a required comparison of surfaces *U*(*х, у*) and *W*(*х, у*) can be realized with a new sectioning method described below. This method includes the following operations:

1. find maximum amplitudes of signals *U*(*х, у*) and *W*(*х, у*) (Figure 1):

$$U_{\text{max}}=\text{max}U\left(x,y\right)=h_{u},$$

$$W_{\text{max}}=\text{max}W\left(x,y\right)=h_{w};$$

2) find values of areas *S*_0,75*u*_ and *S*_0,5*u*_ of sections at levels 0,75 *h*_*u*_ and 0,5 *h*_*u*_ respectively for the signal *U*(*х, у*), and *S*_0,75*w*_ and *S*_0,5*w*_ at levels 0,75 *h*_*w*_ and 0,5 *h*_*w*_ respectively for signal *W*(*х, у*);
3) Calculate approximate values of form factors ${\tilde{r}}_{v,u}$ and ${\tilde{r}}_{v,w}$ for signals *U*(*х, у*) and *W*(*х, у*) respectively;
  1 $$r_{v,u}\approx {\tilde{r}}_{v,u}=r_{s,u}=S_{0,75u}/S_{0,5u},$$
  2 $$r_{v,w}\approx {\tilde{r}}_{v,w}=r_{s,w}=S_{0,75w}/S_{0,5w},$$

where ~ is a sign of the approximate value,

3 $$\begin{array}{cc} r_{v,u}=V_{0,5u}/h_{u}S_{0,5u}, & 0<r_{v,u}\leq 1 \end{array}$$

4 $$\begin{array}{cc} r_{v,w}=V_{0,5w}/h_{w}S_{0,5w}, & 0<r_{v,w}\leq 1 \end{array}$$

*V*_0,5*u*_ and *V*_0,5*w*_ are cumulative values of amplitudes of signals *U*(*х, у*) and *W*(*х, у*), below levels 0,5 *h*_*u*_ and 0,5 *h*_*w*_ respectively;

We will prove that *r*_*v*_ ≈ *r*_*s*_ on the example of the current signal *U*(*х, у*) in the following way.

A volume ${\overset{⌢}{V}}_{0,5u}$ of the figure above the section of its surface *U*(*х, у*) at the level 0,5 *h*_*u*_ may be found by one of the known formulas of the approximate calculation of integrals for equidistant nodes, for instance, by Simpson’s formula (Kendall, 1989):

5 $$\begin{array}{ll} {\overset{⌢}{V}}_{0,5u} & \approx h_{u}[S\left(U_{\text{max}}\right)+4S_{0,75u}+S_{0,5u}]/12\approx \\ \approx h_{u}S_{0,5u}\left(4r_{s,u}+1\right)/12 \end{array}$$

As *S*(*U*_max_) = *S*_1*u*_ is a sectional area of the surface at the level of the maximal amplitude, most often *S*_1*u*_ ≈ 0.

4) compare form factors *r*_*s*, *u*_ and *r*_*s*, *w*_ (instead of the polyelemental comparison of surfaces).

Using (5), one can easily obtain

6 $$V_{0,5u}={\overset{⌢}{V}}_{0,5u}+h_{u}S_{0,5u}/2\approx h_{u}S_{0,5u}\left(4r_{s,u}+7\right)/12$$

from which follows

7 $${\tilde{r}}_{v,u}\approx \left(4r_{s,u}+7\right)/12$$

A linear dependence between factors ${\tilde{r}}_{v}$ and *r*_*s*_ makes it possible to use the factpr *r*_*s*_ as a characteristic of the form of the respective surface.

Possibilities of classification of surface types with the factor *r*_*s*_ are reflected in Table 1. Different values of the factor *r*_*s*_ correspond to different types of surfaces of figures, whose examples presented in the table.

**Table 1:** **Classification of surface types by coefficient** ***r***

Shape of the longitudinal section	Factor values			Inaccuracy ***δr***_***v***_
	***r***_***s***_	${\tilde{r}}_{v}$	***r***_***v***_	
1. Prism, cylinder	1	11/12 ≈ 0.92	1	1/12 ≈ 0.083
2. Pyramid, cone	1/4 = 0.25	2/3 ≈ 0.67	2/3 ≈ 0.67	0
3. Hemisphere	7/12 ≈ 0.58	7/9 ≈ 0.78	7/9 ≈ 0.78	0
4. Hemisphere with a base	3/4 = 0.75	5/6 ≈ 0.83	5/6 ≈ 0.83	0
5. Gaussoid	ln(4/3)/ln2 ≈ 0.415	8.66/12 ≈ 0.722	1/2 ln ≈ 0.721	≈ 0
$\left(1/\sqrt{2\pi }\right)\text{exp}\left\{-\left(x^{2}+y^{2}\right)/2\right\}$				

An important advantage of the factor *r*_*s*_ for some types of surfaces is its independence from the scale coefficient, shift and orientation of the respective surface.

The factor 4*r*_*s*_ characterizes a generalized surface convexity: if 4*r*_*s*_ > 1, the surface is convex; if 4*r*_*s*_ < 1, the surface is flat in the generalized sense; and if 4*r*_*s*_ = 1, the surface is linear in the generalized sense.

It is important to mention that a surface can be characterized by the area of the effective cross-section *S*_*э*_:

8 $$\begin{array}{ll} S_{э}= & 2\;\left(V_{0,5}-S_{0,5}\cdot h\cdot 0,5\right)/h\approx \\ \approx 2\;\left(S_{0,5}{\tilde{r}}_{v}h-S_{0,5}\cdot h\cdot 0,5\right)/h=2S_{0,5}({\tilde{r}}_{v}-0,5) \end{array},$$

The sectioning method may be used for a laser beam signal sample, and the shape coefficient may be used as a sample parameter. This method will be used to determine laser beam centers.

9 $$S_{э}\;\approx \;{\tilde{S}}_{э}=\left\{\begin{array}{c} \left(4S_{0,75}+S_{0,5}\right)/6\;\mathrm{if}\;S_{1}\;\approx \;0,\\ \left(S_{1}+4S_{0,75}+S_{0,5}\right)/6\;\mathrm{if}\;S_{1}\;\neq \;0, \end{array}\right.$$

where *S*_1_ is a sectional area of the signal on its maximal level.

### The algorithm of control of the laser beam spot shape and experimental results

An algorithm of the method for a frame of 128∗128 decomposition elements (DE further on) is the following:

1. Find a point with the maximum brightness W_max_ on the image;
2. Find values of brightness:
  $$\begin{array}{ccc} w_{0.75}=W_{\text{max}}*0,75 & \text{and} & w_{0,5=}0,5*W_{\text{max}} \end{array}.$$
3. Find areas of surfaces S_0.75_ and S_0.5_:
  10 $$\begin{array}{c} S_{0.75}=\sum_{x=0}^{N-1}\sum_{y=0}^{N-1}\left\{\begin{array}{c} S_{0.75}+1,w(f\left(x,y\right)\geq w_{0.75}\\ S_{0.75},w(f\left(x,y\right)<w_{0.75} \end{array},\right.\\ S_{0.5}=\sum_{x=0}^{N-1}\sum_{y=0}^{N-1}\left\{\begin{array}{c} S_{0.5}+1,w(f\left(x,y\right)\geq w_{0.5}\\ S_{0.5},w(f\left(x,y\right)<w_{0.5} \end{array},\right. \end{array}$$

where *N* is a number of decomposition elements in the frame line (column) equal to 128, W(f(x,y)) is a brightness value of the point.

4) Then we will find form factors *r*:
  11 $$r=\frac{S0.75}{S0.5}$$

It was found out experimentally, that an image is “good” if 0.7 < *r* <0.8; if not, go to step 1.

5) Find image centers (González and Woods, 2003):
  12 $$\begin{array}{l} x=\frac{1}{M}\sum_{x=0}^{N-1}\sum_{y=0}^{N-1}w\left(f\left(x,y\right)\right)*x,\\ y=\frac{1}{M}\sum_{x=0}^{N-1}\sum_{y=0}^{N-1}w\left(f\left(x,y\right)\right)*y,\\ M=\sum_{x=0}^{N-1}\sum_{y=0}^{N-1}w\left(f\left(x,y\right)\right), \end{array}$$

where x, y are values of x and y coordinates respectively; w(f(x,y)) is the point brightness value; М is the image “mass” (a sum total of brightnesses of all points of the image).

**Figure 2:**
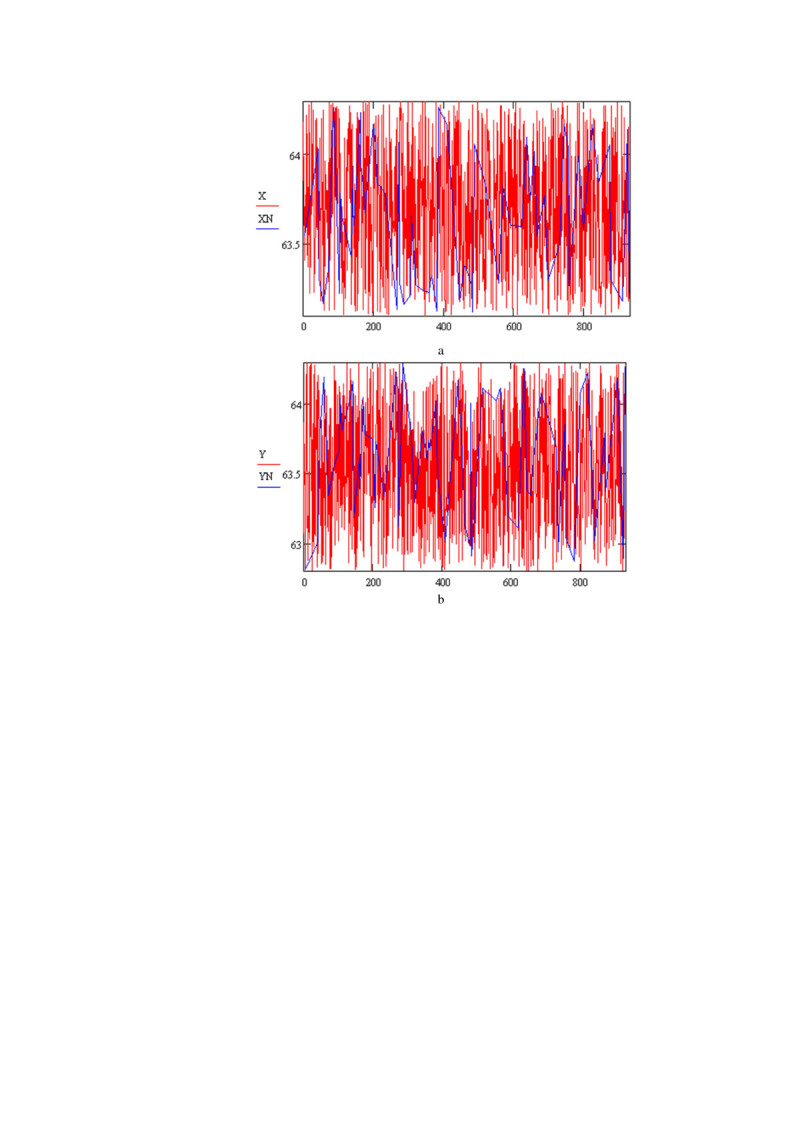
**A distribution of coordinates of laser beam centers. (a)** X - value and **(b)** Y - value of centers (where X and Y are coordinates of the whole laser beam route respectively, XN, YN are coordinates of spot centers after sampling).

The following operations are performed during the investigation of the laser beam route:

An initial sample is formed of 10% laser beam spot images. During its formation, tunnel boundaries are determined with the enumerative technique. After that, sampling of laser beam spot images is conducted, and a group of “good” images is determined (Figure 2).

15 laser beam routes were studied (Basov et al., 1998), and the following results were obtained (presented for two routes):

Tunnel boundaries a = 0.77, b = 0.7. Those values are determined by analyzing a training sample with a method of optimization immediately before running the algorithm.

Difference between the maximum and minimum values of coordinates is 1.147.

## Modeling of the sectioning method for classification of laser beam spots based on the PLD

The algorithm described in The algorithm of control of the laser beam spot shape and experimental results section is relatively simple but has high performance, which allows to use it in add-in systems. The algorithm was used for the first time for this problem based on the Texas Instruments signal processor TMS320C5510 (Timchenko and Kutaev, 2002) with clock frequency of 200 MHz; it takes 10 ms to process one image. About 130 commands are used per one decomposition element.

At the same time, the following drawbacks were revealed in the process of operation: a high enough frequency presents increased requirements to the electromagnetic compatibility and a level of unit realization; almost all processor time is used for image processing, which does not allow to use the processor for other operations.

In addition, at present, video matrixes with higher resolution may be used to increase image refinement. Processing time of one image with the same process for work, for instance, with a matrix of 256 by 256 decomposition elements, may be calculated by the formula:

13 $$\tau =\frac{1}{F}*\sum_{i=1}^{k}n_{i},$$

where *F* is the processor frequency of 200 MHz, *k* = 130 is a number of commands per decomposition element, *n* is a number of decomposition elements in a frame (256 by 256 DE = 65536 DE), i.e. processing of one frame takes:

$$\tau =\frac{1}{2*10^{8}}*130*65536=42,5*{10^{-2}}_{\;\mathrm{MS}.}$$

Processing time *τ* indicates that the processor frequency is not sufficient for the real-time processing and requires a four times more powerful processor. And in case of an even bigger matrix, a need in increased performance grows exponentially.

### Development of a parallel algorithm for the sectioning method

The following conclusions can be made after a more detailed study of the algorithm:

1. Most operations used in the algorithm are simple mathematical operations (multiplication and addition, comparison and counting).
2. Some formulas use the same variables (for instance, *w*(*f*(*x*, *y*)) in formulas 1 and 3), which provides an opportunity of their sharing.

Hence, a conclusion can be made about a possibility to use the computational hardware developed especially for the algorithm realized, which would allow building the parallel computational structures. The most flexible tool for that are the programming logic devices (PLDs).

**Figure 3:**
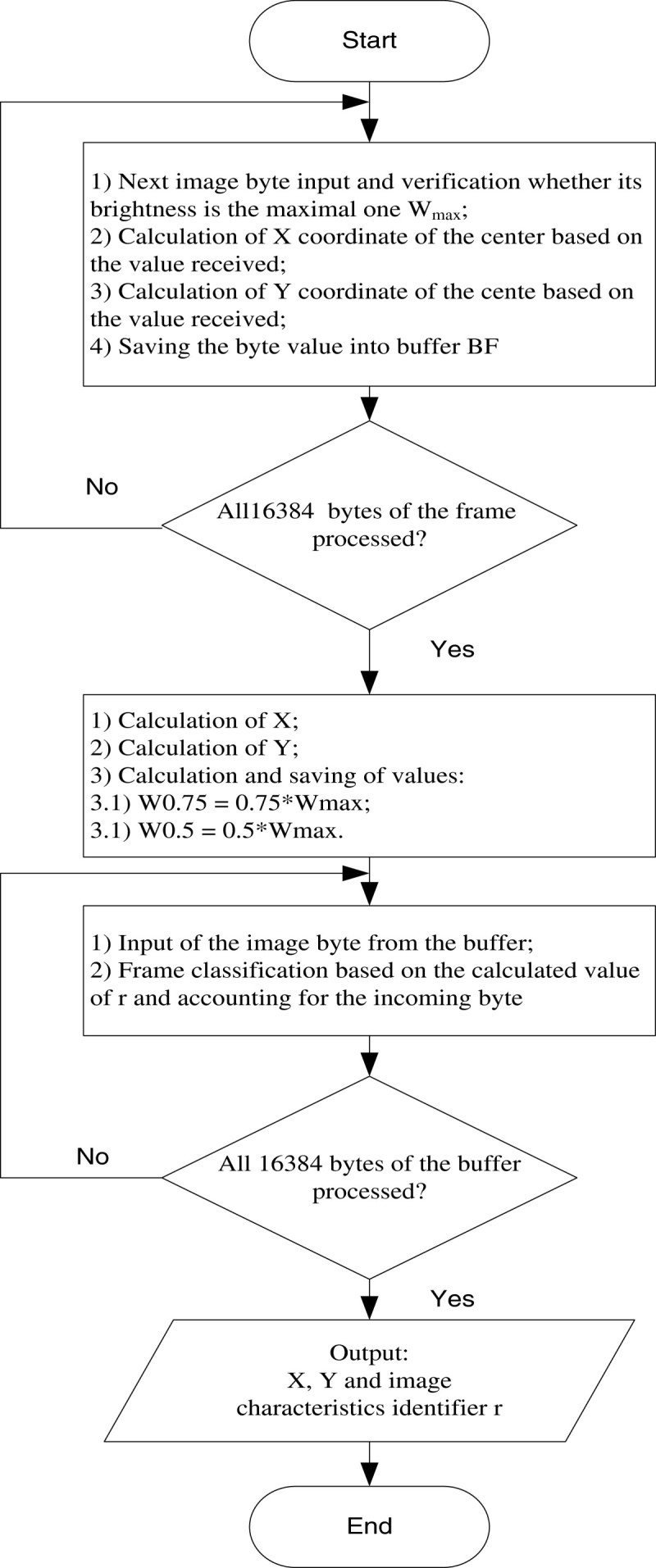
**Flowchart of the parallel algorithm of image processing.**

A parallel algorithm of position determination and classification of laser beam spot images is presented in Figure 3.

Though the flowchart is consequential, operations in each computational unit are performed simultaneously, which results in time-saving in performing operations of the same type.

In the process of development of this algorithm, an order of some operations was changed in comparison with the consequential algorithm. For instance, according to step 4 of the algorithm, a form factor is calculated, and then on its basis a decision about the position computation is made. Instead, in the parallel algorithm, the position is calculated simultaneously with finding a decomposition element with the maximum brightness W_max_. This is explained by the fact that in the PLD, unlike the traditional programming, algorithm elements occupy the circuit space regardless of whether they will be performed at a certain stage or not (Wakerly, 1999). In addition, this technique allows to decrease a number of iterations performed over one image frame.

**Figure 4:**
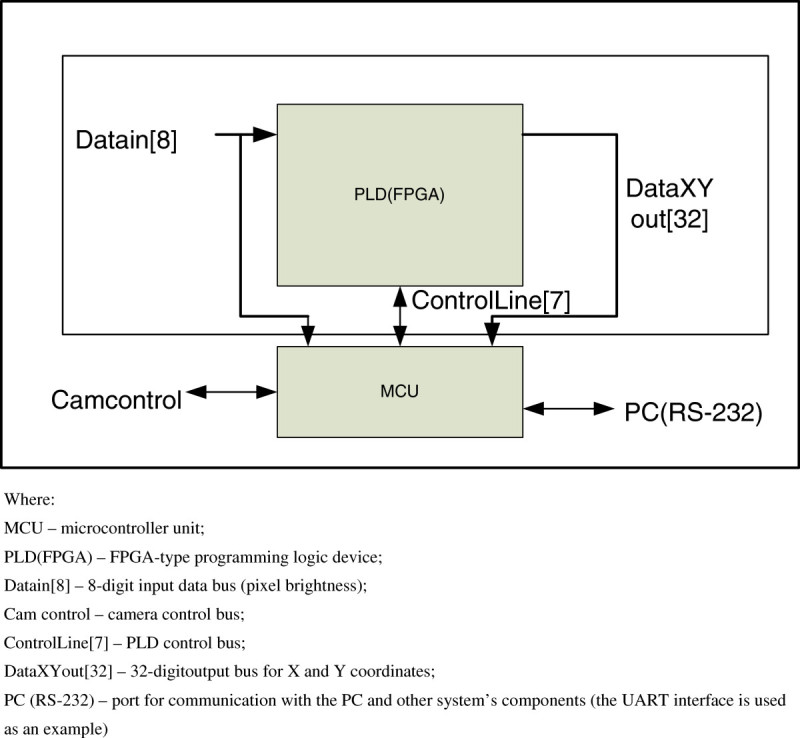
**Block diagram of the image processing and classification device.**

For the simplicity of implementation, the algorithm implies a PLC-based computational part of the schematic, and the computational process is controlled by means of an external controller or an embedded controller kernel of PLD (Altera, 2011). A block diagram of the device is presented in Figure 4.

In this schematic, a microcontroller unit is controlling a camera, the PLD and PC communication, and the PLD is occupied with calculations of coordinates and image characteristics.

### The PLD-based modeling of the parallel algorithm of the sectioning method

According to the parallel algorithm, a schematic of the image control and processing unit should meet the following requirements:

1. be friendly to upgrades depending on the video matrix used;
2. have a module structure for controlling individual units and maximally reduce the operation time.

**Figure 5:**
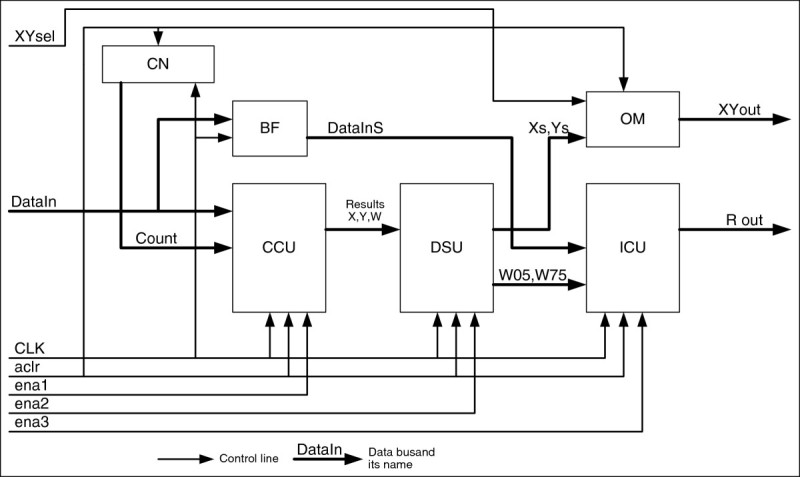
**Block diagram of the PLD module.**

Those requirements are satisfied by a schematic presented in Figure 5 and developed on the basis of the Quartus II 10.1 software by *Altera*, one of the world leaders in PLD production. The software uses a graphics builder and VHDL hardware description language (Ashenden, 1995) in respect to possessing of 128 by 128 DE frames. At the same time, usage of registers with excessive capacity allows to apply it to bigger matrixes as well without significant alterations.

The device consists of the following blocks:

The coordinate calculation unit (CCU) calculates values of X and Y and the maximal brightness W_max_; the counter (CN) is used to submit input byte coordinates (its number) to the CCU; the data storage unit (DSU) stores intermediate values of X and Y and values of W_0,5_ (W0.5) and W_0,75_ (W0.75), the image classification unit (ICU) determines a form factor *r* (classifies images as “good” or “bad”); the 16384-byte circular buffer (BF) is used to store frame data during the circuit operation; the output multiplexor (OM) is an asynchronous multiplexor enabling a serial output of X or Y coordinates.

External buses of the circuit are presented in Table 2:

**Table 2:** **Buses of the PLD module**

Name	Direction	Comment
Aclr	In	Asynchronous clear input;
CLK	In	Clock input;
Ena1	In	CCU clock enable input;
Ena2	In	DSU clock enable;
Ena3	In	ICU clock enable;
Rpin	Out	“Good”/“bad” image indication output
Datain[8]	In	Input data bus;
Dataxy[32]	Out	Х and Y output data bus;
XYsel	In	Selection of the output coordinate: «0»- Х, «1»-Y;

The circuit works in the following way:

After a signal “1” arrives at the bus *aclr*, all registers are cleared, and a circuit is converted into an initial state. After that, a signal of logical “1” is sent to the bus input *ena1* (it also gets to the BF storage input); a data byte is delivered to the input *Datain*; then a clock pulse passes. A value of the counter CN increases at the clock pulse edge, and the data is recorded in the buffer BF. The operation is repeated for 16384 clock pulses until the processing of the frame with the dimensionality of 128 by 128 DE is completed, and values of X and Y coordinates and of the maximal brightness W appear at the CCU output therewith.

At the next step, a signal of logical ‘1’ is fed to the *ena2* input, and a signal of logical ‘0’ is fed to the *ena1* input; then a clock signal CLK arrives; as a result, data for X, Y and W are recorded for storage in the DSU.

The third step includes nulling of *ena1* and *ena2* and feeding a signal of logical ‘1’ to the *ena3* input; then 16384 clock pulses arrive to the CLK input. As a result, comparison of brightness of the individual byte with values of half (W05) and ¾ (W075) of maximal brightness takes place in the ICU, and areas of respective sections are determined. If their dividend (form factor *r*) falls within permissible limits, the image is considered “good”. To save time during this unit’s operation, values of X and Y may be obtained from the output *DataXY* using a control output *XYsel* (logic ‘0’ signal means value X, ‘1’ – value Y respectively).

After that, an asynchronous reset should be performed, and the system is ready to process a new image. Therefore, the image processing time is 32770 clock pulses.

**Figure 6:**
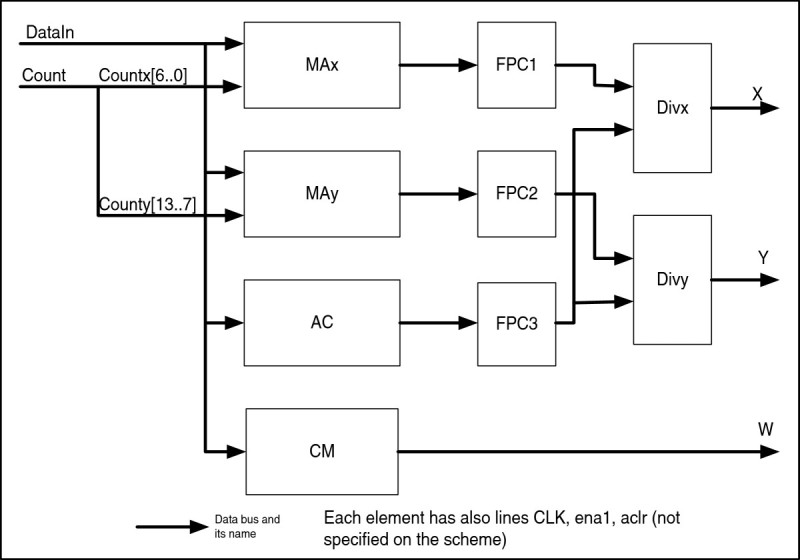
**Coordinate calculation unit.**

Let us consider individual units of the schematic in more details. The coordinate calculation unit CCU is presented in Figure 6, and functions of its inputs are presented in Table 3.

**Table 3:** **Signals of the CCU**

Name	Direction	Comment
Aclr	In	Asynchronous clear input;
CLK	In	Clock input;
Ena1	In	Clock enable input
DataIn	In	Input data bus;
Countx[6..0]	In	Counter input bus for X
County[13..7]	In	Counter input bus for Y
X[32]	Out	Output of X value;
Y[32]	Out	Output of Y value;
W[8]	Out	Output of W value;

The unit consists of the following elements:

MAx, MAy – multiplier-accumulators for X and Y coordinates respectively, they store all values for x and y; AC – accumulator of weight values (ΣW); CM – comparator and 8-digit register – a circuit of selection and storage of the maximal brightness point; FPC1-3 (code converter) – converters of the 32-digit unsigned integer into a 32-digit floating-point number; DIVх, DIVу (X and Y dividers respectively) – 32-digit dividers of floating-point numbers.

After a general reset, logical “0” is set up in registers of all elements, then the byte appears at the input *DataIn*, and after the clock pulse is received, its value is sent to the inputs *Max*, where it is multiplied by the counter value. After that, the result obtained is accumulated in the accumulator. Data for Y coordinate are processed similarly, and for W data a simple addition with accumulation is realized. Then obtained sums arrive to dividers *Divх*, *Divy* through the code converter (FPC). At the same time, the CM circuit compares value W of the previous byte with its current value, and if the new value is bigger, it is being registered in the register. As we stated above, one frame of 128 by 128 DE requires 16384 clock pulses. As a result, values of X, Y and W appear on the outputs.

The data storage unit (DSU) is a simple register where intermediate data is stored.

**Figure 7:**
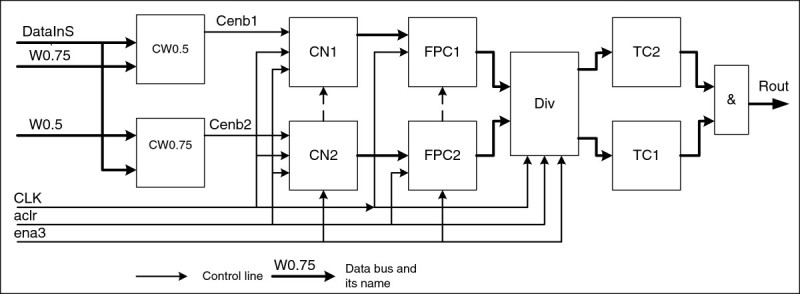
**Image classification unit.**

The image classification unit (ICU) is the most complicated element (Figure 7). This unit is designed for calculation of the form factor *r*, i.e. for image classification into “good”, with *r* = 1, and “bad”, with *r* = 0 respectively. The unit consists of the following elements and connections (Table 4).

**Table 4:** **Signals of the ICU**

Name	Direction	Comment
Aclr	In	Asynchronous clear input;
CLK	In	Clock input;
Ena3	In	Clock enable input;
DataInS[8]	In	Input data bus (from buffer)
W0.5	In	Input bus W05;
W0.75	In	Input bus W075;
Rout	Out	“Good”/“bad” image indication output

The СW0.5, СW0.75 are comparators that compare incoming frame byte with respective values of brightness; counters CN1, CN2 count a number of points above a respective brightness threshold; code converters FPC1, FPC2 transform an integer into a floating-point number; Div is a divider of floating-point numbers received from CN1 and CN2; threshold comparators TC1 and TC2 compare values received from the Div with preset constants (TC1 for r = 0.7 and TC2 for r = 0.8).

The circuit works in the following way:

When an input byte arrives, comparators СW0.5 and СW0.75 produce authorization signals for their counters. On arrival of the clock pulse, counters increase their indications by one subject to the authorizing signal of the comparator (СW0.5, СW0.75). This operation is repeated for 16384 times for all frame decomposition elements. Then counter indications are transformed into floating-points numbers, and an operation of their division is performed in the *Div*. The number obtained is compared in the threshold comparators TC1 and TC2 with constants. If this number is within the limits, logical “1” will appear at their outputs, which send a signal of logical “1” through the element “&” to the output, therefore signaling about a “good” image.

The circuit was modeled in the media ModelSim Altera 6.6. on the basis of the Cyclone II microchip of EP2C20Q240C8 type. As a result, the following results were obtained:

Number of logic elements – 2,454/18,752 (13%).

Number of pins used – 47.

*f*_*max*_ – 112 MHz.

Therefore, a microchip with a lower capacity may be used for the real device.

**Figure 8:**
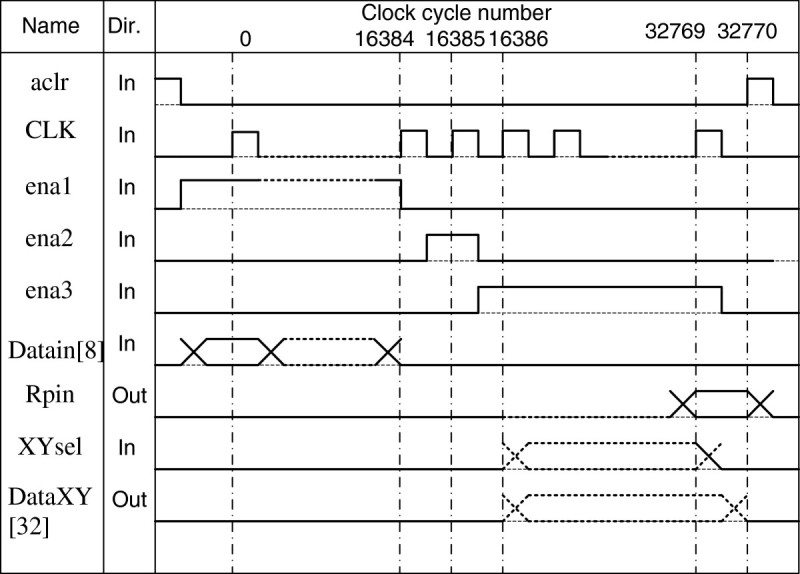
**PLD timing diagram.**

A timing diagram of the PLD unit is presented in Figure 8.

## Discussion of the obtained results

Experiments demonstrate that due to various destabilizing factors, coordinates of energy centers of laser beam spots cannot be measured accurately; however, accuracy can be substantially increased by using a calculation of the form factor of images with their further classification into “good” and “bad”.

According to the suggested sectioning method, the comparison of surfaces is reduced to the comparison of their form factors and does not require their comparison by element with consideration of all cases of difference in their types, scale coefficient, relative shift and turn in space. It is important to mention that equality of form factors of surface, in the general case, allows to attribute them to the same generalized surface type or to approximate surfaces with that type.

Obviously, the sectioning method can easily be extended to the case of the increased number of equidistant sections of the surface, of changes in values of section levels, changes of weight coefficients in areas of sections. In this case, it also makes sense to use a suitable formula of approximate calculation of integrals.

The sectioning method discussed is also promising for the usage in problems of real-time image classification and archiving. An important advantage of this method is simplicity of its realization in terms of both software and hardware.

A conclusion can be made from the diagram (Figure 8) that the schematic is completely static, and that its operation requires (2N + 2) clock pulses to process an image (where N is a number of decomposition elements), knowing that the initial circuit uses *τ* = 10 ms per frame. A required PLD frequency may be calculated:

14 $$f=\frac{\left(2N+2\right)}{\tau };$$

$$f=\frac{\left(2*16834+2\right)}{0.01}=3.27\;\mathrm{MHz},$$

As the maximum chip frequency is 100 MHz, it can be used for matrixes of up to 512∗512 DE without changing a chip for a more expensive and high-performance one.

Therefore, this schematic may be used to improve characteristics of laser transfer systems of various types. A flexibility of PLD programming allows to create such subsystem with a single circuit solution, and characteristics of image processing may be changed through reprogramming the PLD for a target transfer system.

In addition, a relatively small space used by the circuit developed in relation to the capacity of PLD logic cells allows making a conclusion that a research should be conducted on embedding one of broadly available chip architectures. This would allow to remove the microcontroller (Figure 4) from the circuit, thus making it even simpler.

The circuit developed may also serve as a basis for developing subsystems of forecasting characteristics of spot images, which becomes more and more needed with a growth in speed and distance between the transfer systems.

## Conclusions

1. A sectioning method for classification of laser beam spots was developed, which does not require performing time-consuming calculations.
2. A sectioning method-based parallel algorithm developed meets conditions by a number and complexity of operations.
3. The image processing and classification device allows to release the main processor from making one-type operations.
4. The working frequency of the circuit in its basic is 40 times lower than with the use of the digital transfer system, which allows to reduce requirements by the electromagnetic compatibility.
5. The processing device design is modular, which makes it possible to use it for matrixes with higher resolution.

## Authors’ original submitted files for images

Below are the links to the authors’ original submitted files for images. Authors’ original file for figure 1 Authors’ original file for figure 2 Authors’ original file for figure 3 Authors’ original file for figure 4 Authors’ original file for figure 5 Authors’ original file for figure 6 Authors’ original file for figure 7 Authors’ original file for figure 8
