# Supplementary figures and images for: Modeling of the high-performance PLD-based sectioning method for classification of the shape of optical object images
Source: Springerplus. 2013 Dec 27;2(1):692. doi: 10.1186/2193-1801-2-692 (PMC3884084; doi:10.1186/2193-1801-2-692)

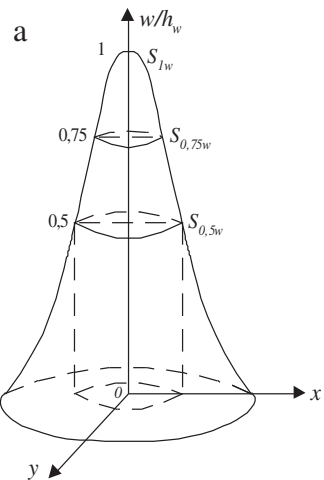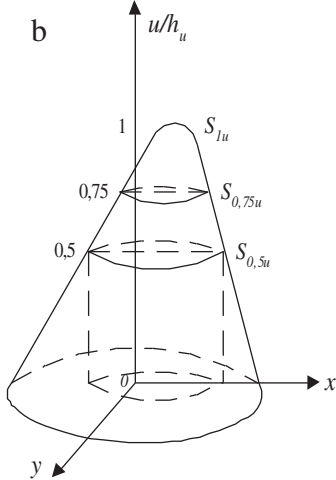

Supplement: Supplementary file 1 — Authors’ original file for figure 1 [file 40064_2013_757_MOESM1_ESM.pdf]

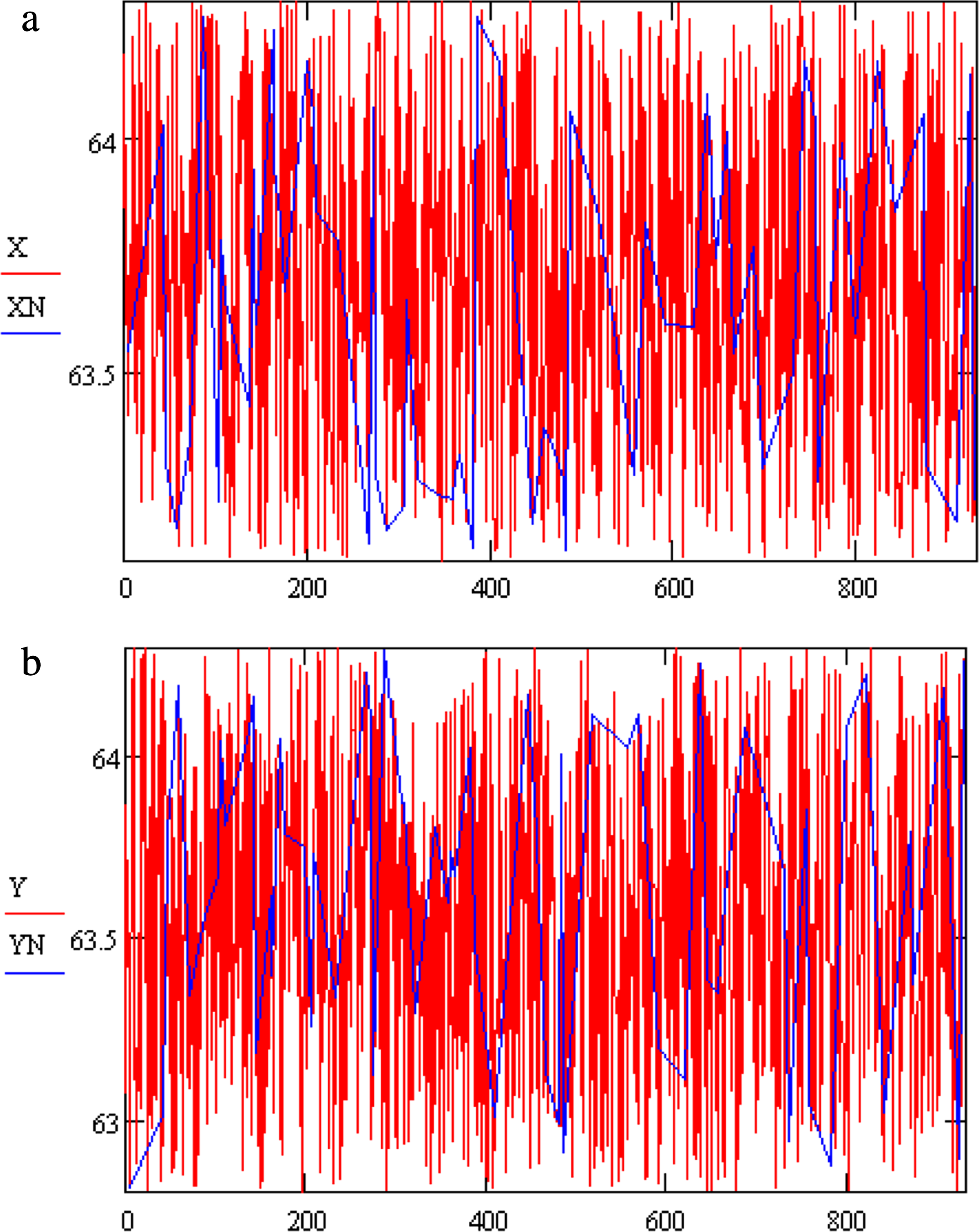

Supplement: Supplementary file 2 — Authors’ original file for figure 2 [file 40064_2013_757_MOESM2_ESM.tif]

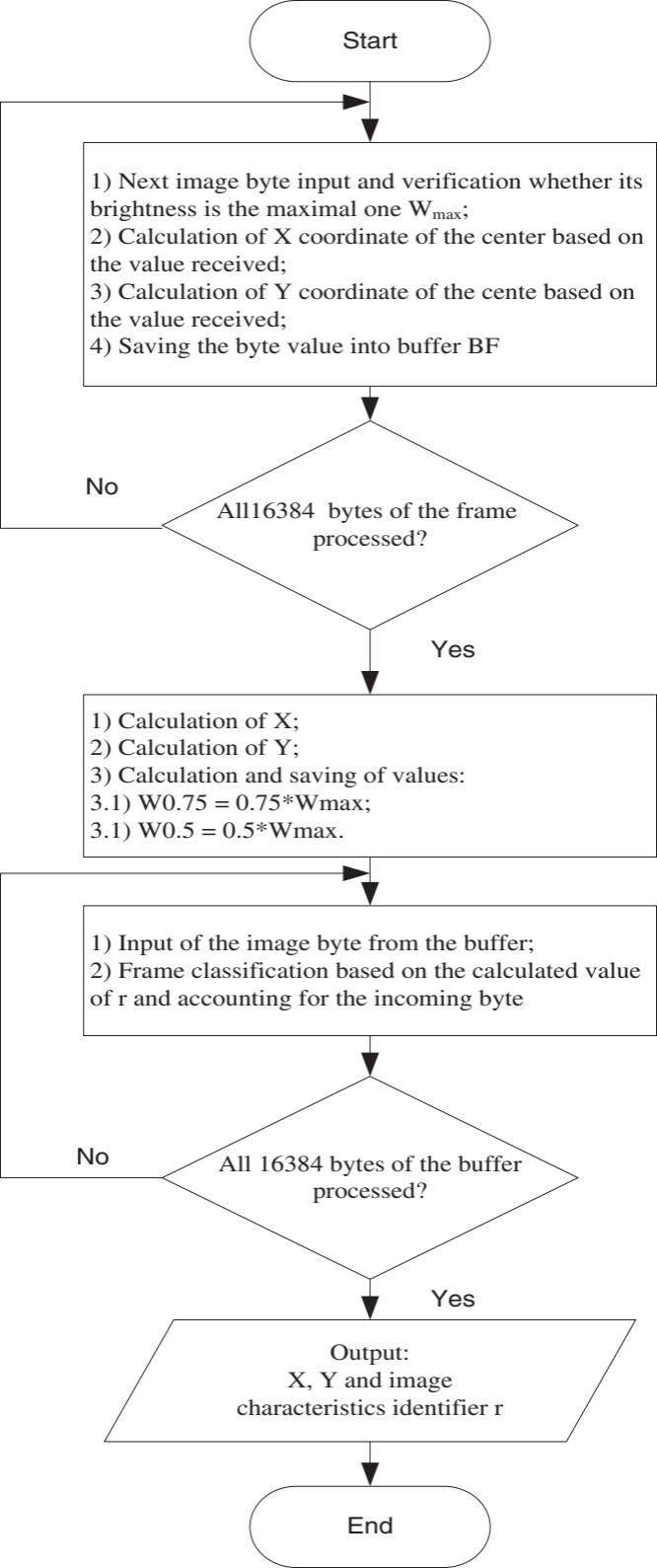

Supplement: Supplementary file 3 — Authors’ original file for figure 3 [file 40064_2013_757_MOESM3_ESM.pdf]

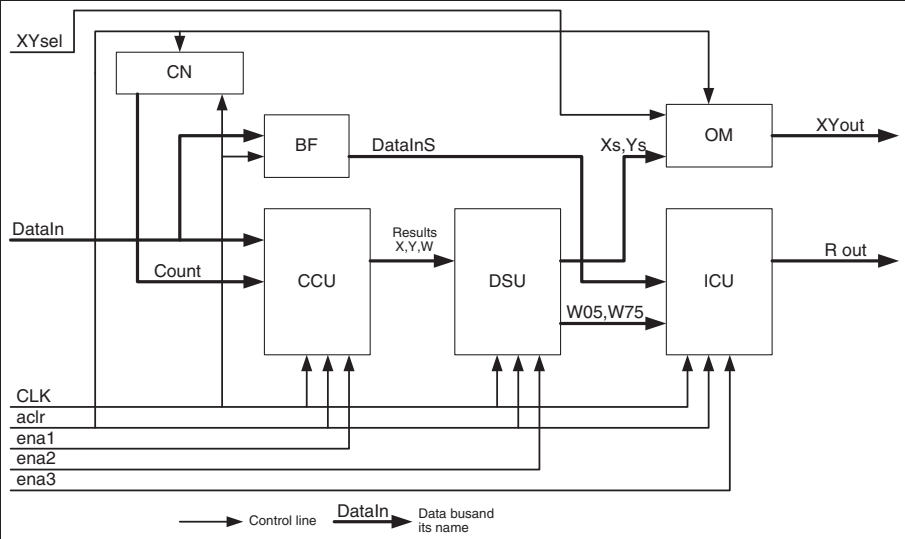

Supplement: Supplementary file 5 — Authors’ original file for figure 5 [file 40064_2013_757_MOESM5_ESM.pdf]

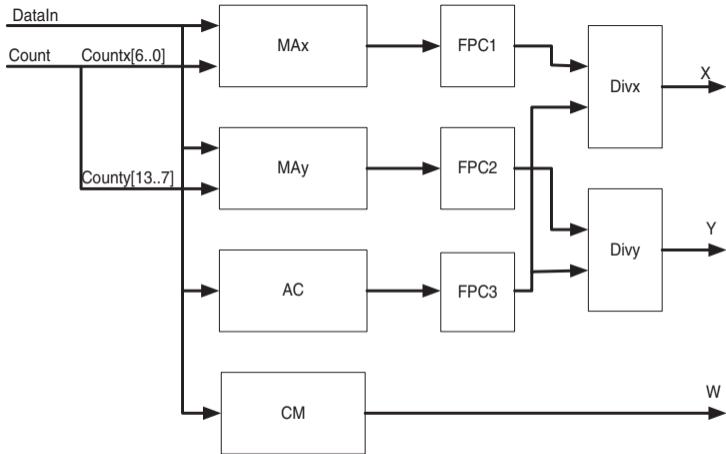

→ Data bus and  
its name

Each element has also lines CLK, ena1, aclr (not  
specified on the scheme)

Supplement: Supplementary file 6 — Authors’ original file for figure 6 [file 40064_2013_757_MOESM6_ESM.pdf]

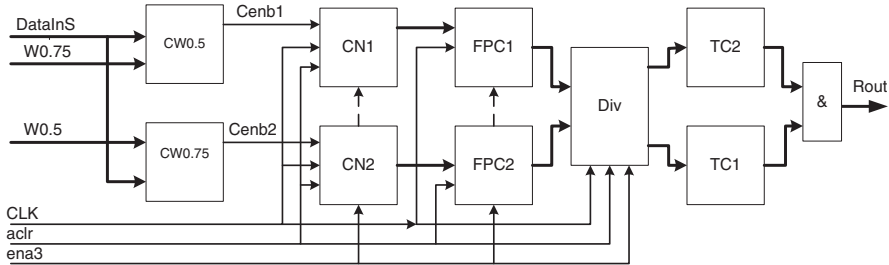

Supplement: Supplementary file 7 — Authors’ original file for figure 7 [file 40064_2013_757_MOESM7_ESM.pdf]

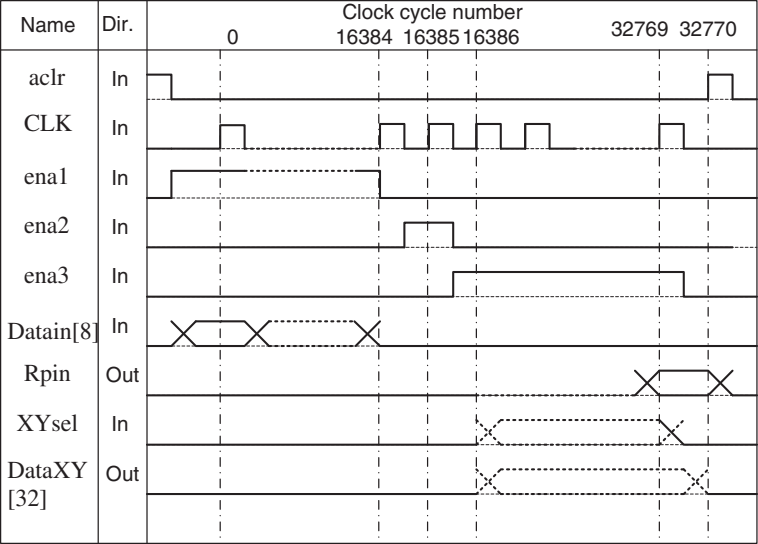

Supplement: Supplementary file 8 — Authors’ original file for figure 8 [file 40064_2013_757_MOESM8_ESM.pdf]
