# Supplementary material for: Modeling of the high-performance PLD-based sectioning method for classification of the shape of optical object images
Source: Springerplus. 2013 Dec 27;2(1):692. doi: 10.1186/2193-1801-2-692 (PMC3884084; doi:10.1186/2193-1801-2-692)
Supplement: Supplementary file 4 — Authors’ original file for figure 4 [file 40064_2013_757_MOESM4_ESM.pdf]

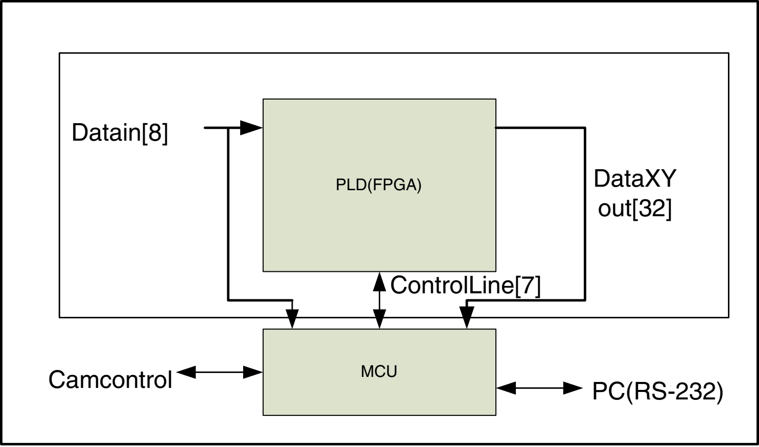

Where:

MCU – microcontroller unit;

PLD(FPGA) – FPGA-type programming logic device;

Datain[8] – 8-digit input data bus (pixel brightness);

Cam control – camera control bus;

ControlLine[7] – PLD control bus;

DataXYout[32] – 32-digitoutput bus for X and Y coordinates;

PC (RS-232) – port for communication with the PC and other system's components (the UART interface is used as an example)
